# *Cannabis sativa* L. roots extract modulates gastrointestinal motility and ameliorates ethanol-induced gastric ulcers in animal models

**DOI:** 10.3389/fphar.2026.1743428

**Published:** 2026-01-30

**Authors:** Pedro Guilherme Sousa de Sá, João Gabriel de Souza Rocha, Juliane Maria dos Santos Silva, Nathália Andrezza Carvalho de Souza, Tarcísio Cícero de Lima Araújo, Victória Laysna dos Anjos Santos, Pedro Modesto Nascimento Menezes, Raimundo Campos Palheta Junior, Fabrício Souza Silva, Larissa Araújo Rolim

**Affiliations:** 1 Graduate Program in Biotechnology, Northeast Biotechnology Network (RENORBIO), Rural Federal University of Pernambuco (UFRPE), Recife, Brazil; 2 Drugs, Medicines and Food Analysis Center (CAFMA), Federal University of Vale do São Francisco (UNIVASF), Petrolina, Brazil; 3 Laboratory of Experimental Pharmacology (LAFEX), Federal University of Vale do São Francisco (UNIVASF), Petrolina, Brazil; 4 Laboratory of Veterinary Pharmacology, Department of Veterinary Medicine, Federal University of Vale do São Francisco (UNIVASF), Petrolina, Brazil

**Keywords:** antibacterial, Cannabis sativa, diarrhea, gastric ulcer, gastrointestinal motility, terpenes

## Abstract

**Introduction:**

*Cannabis sativa* L. roots are traditionally used to manage gastrointestinal (GI) disorders; however, experimental pharmacological evidence supporting these uses remains limited. This study investigated the chemical profile, safety, and GI-related pharmacological effects of an ethanolic extract of *C. sativa* roots (CEECs).

**Methods:**

Chemical characterization was performed by spectrophotometric determination of total triterpenes and HPLC profiling. Safety and pharmacological effects were assessed through acute oral toxicity testing, antibacterial assays, and in vivo murine models of gastric emptying, diarrhea, and ethanol-induced gastric ulcer.

**Results:**

CEECs showed a total triterpene content of 67.64 ± 5.39 μg LE·mg^−1^, and HPLC analysis detected *p*-coumaric acid and N-*trans*-feruloyltyramine. In vivo, CEECs significantly delayed gastric emptying at 50 mg·kg^−1^ (*P* = 0.0033) and reduced fecal output in the castor oil-induced diarrhea model at 50 (*P* < 0.001) and 100 mg·kg^−1^ (*P* = 0.0233), with no effect in the magnesium sulfate-induced model. CEECs also significantly reduced ethanol-induced gastric mucosal injury at 50 mg·kg^−1^ (*P* = 0.0484) and 100 mg·kg^−1^ (*P* = 0.0164). No signs of acute toxicity were observed at 2000 mg·kg^−1^. Antibacterial activity against Staphylococcus aureus strains was weak under the tested conditions.

**Discussion:**

These findings provide experimental support for the traditional use of *C. sativa* roots in GI disorders and indicate their potential as a non-psychoactive source of bioactive constituents.

## 1 Introduction

Gastrointestinal (GI) disorders represent a major public health challenge worldwide due to their high morbidity, mortality, and economic burden (Gikas and Triantafillidis, 2014; Peery et al., 2022). Across different cultural and historical contexts, medicinal plants have played a central role in the management of GI symptoms, and *Cannabis sativa* L. has been repeatedly cited in traditional medical systems for treating diarrhea, gastritis, dysentery, and other digestive disturbances (Di Marzo and Piscitelli, 2011; Thapa et al., 2023). These records suggest a long-standing empirical association between cannabis use and gastrointestinal health.

In contemporary clinical practice, however, cannabis-based medicines are primarily derived from the plant’s aerial parts and are indicated mainly for chemotherapy-induced nausea and vomiting or appetite stimulation. These effects are attributed to phytocannabinoids acting through the endocannabinoid system (ECS), a regulatory network widely expressed throughout the GI tract and involved in the modulation of motility, secretion, and epithelial function (Kumar et al., 2021; Legare et al., 2022). Consequently, pharmacological research has largely focused on cannabinoid-rich tissues, while other plant parts have received comparatively less attention.

In contrast, the roots of *C. sativa*, despite being frequently mentioned in ethnomedical records, remain poorly explored from a pharmacological perspective and are often discarded as agricultural by-products. Ethnopharmacological surveys from South and Southeastern Asia describe the use of cannabis roots in the treatment of diarrhea, constipation, dyspepsia, and inflammatory conditions (Miano et al., 2011; Elhendawy et al., 2018; Altaf et al., 2019; Saha et al., 2019; Kornpointner et al., 2021; Odieka et al., 2022). However, these traditional applications have received little experimental validation.

Notably, although cannabis roots contain only trace amounts of cannabinoids, they are enriched in other classes of secondary metabolites, including triterpenoids and phenolic compounds, which may exert biological effects relevant to gastrointestinal function (Silva et al., 2014; Elhendawy et al., 2018; Kornpointner et al., 2021; Lima et al., 2021; Huang et al., 2023). While previous studies have described antibacterial properties of *C. sativa* root extracts, systematic investigations addressing their effects on gastrointestinal motility, diarrhea, and mucosal integrity are currently lacking. The absence of integrated pharmacological data limits the interpretation of their traditional use and hinders the identification of chemical markers to support future ethnopharmacological investigations.

To address this gap, the present study investigated the pharmacological effects of an ethanolic extract of *C. sativa* roots (CEECs) cultivated in São Francisco Valley, northeastern Brazil. Chemical and biological assays were performed, including chromatographic profiling, total triterpene quantification, antibacterial evaluation, and *in vivo* models of gastric emptying, diarrhea, and ethanol-induced gastric ulcers. The results provide experimental evidence supporting the traditional use of cannabis roots in GI disorders and contribute to the identification of pharmacologically relevant chemical markers.

## 2 Materials and methods

### 2.1 Plant material

Samples of *Cannabis sativa* L. (plant name verified by MPNS, 2025) were obtained from the Federal Police of Brazil under official authorization, following seizure of illegal cultivation sites in the São Francisco Valley (Pernambuco and Bahia states, Brazil). The plant identity was confirmed by comparison with a voucher specimen (no. 23331) deposited in the Vale do São Francisco Herbarium, Federal University of Vale do São Francisco (Petrolina, Pernambuco, Brazil). The study was authorized by the Brazilian Federal Court (case no. ALE.Pje.0017.0001/2016), the Brazilian Health Regulatory Agency (ANVISA, Authorization no. 016/2016), and the National System for the Management of Genetic Heritage and Associated Traditional Knowledge (SISGEN, registration no. AC1D0D9).

### 2.2 Processing of plant material and extract preparation

Processing of the plant material followed the method described by Araújo et al. (2024). *C. sativa* roots were collected and processed as obtained, without anatomical subdivision or selection of specific portions of the root system. The roots were cleaned and sanitized, then dried at 45 °C for 3 days and pulverized using a knife mill. The resulting root powder was stored at room temperature and protected from light.

A 200 g portion of the powder was macerated at room temperature in 2 L of absolute ethanol, with solvent renewal every 3 days, until exhaustion of soluble compounds was achieved, as indicated by the absence of color change in the solvent. The combined ethanolic extracts were concentrated under reduced pressure using a rotary evaporator, yielding the crude ethanolic extract of *C. sativa* roots (CEECs).

### 2.3 Spectrophotometric quantification of total triterpenes

The spectrophotometric quantification of total triterpenes was performed according to the method described by Pedrosa et al. (2020). A 75 µL aliquot of CEECs stock solution at 1 mg mL^-1^ in ethanol was evaporated at 85 °C. Subsequently, 250 µL of vanillin solution at 50 mg mL^-1^ and 500 µL of sulfuric acid were added. The reaction mixture was heated at 60 °C for 30 min, cooled in an ice bath, and treated with 2.5 mL of acetic acid. After 20 min of cooling, the solution was maintained at room temperature. The blank solution was prepared using the same procedure, with ethanol replacing CEECs solution.

A calibration curve ranging from 3.077 to 24.615 μg mL^-1^ was constructed using lupeol as the reference standard, showing linearity described by the equation *y = 0.0837x – 0.1122* with a coefficient of determination R^2^ = 0.9966. Absorbance was measured at 548 nm in a UV-Vis spectrophotometer. The total triterpene content was expressed as micrograms of lupeol equivalents per milligram of extract (µg LE∙mg^-1^). All measurements were performed in triplicate.

### 2.4 High performance liquid chromatography (HPLC) analysis of CEECs

#### 2.4.1 Chromatographic apparatus

HPLC analysis was carried out using a Shimadzu Prominence series LC20-AT system equipped with a DGU-20A5 degasser, a SIL-20A HT autosampler, a CTO-20A column oven, an SPD-M20A photodiode-array detector (PDA), and a CBM-20A communication bus module.

#### 2.4.2 Chromatographic conditions

The analysis followed the method described by Araújo et al. (2024) with minor modifications. Separation was performed in a Shimadzu Shim-pack CLC-ODS(M) C18 reversed-phase column (250 mm × 4.6 mm, 5 μm) maintained at 30 °C under gradient elution. The mobile phase consisted of solvent A (0.1% formic acid in ultrapure water) and B (acetonitrile) according to the following program: 0–45 min, 15%–60% B. The flow rate was set to 1.0 mL min^-1^, and injection volumes were 10 µL for CEECs solution and 5 µL for reference standards. Detection was performed was performed using the PDA, with chromatograms monitored at 280 nm and ultraviolet spectra recorded over the full acquisition range for peak characterization and comparison with reference standards.

#### 2.4.3 Samples

A CEECs sample was prepared by dissolving the material in methanol to obtain a final concentration of 5 mg mL^-1^. The solution was filtered through a 0.22 µm PVDF syringe filter and transferred to amber HPLC vials prior to analysis. Reference standards were prepared and analyzed under identical chromatographic to allow comparison of retention times and ultraviolet absorption profiles.

Chromatographic peaks were integrated at 280 nm. Relative peak areas were calculated by normalizing individual peak areas to the total integrated chromatographic area and expressed as percentage values.

### 2.5 Animals

Swiss mice (*Mus musculus*) of both sexes, weighting 30–40 g and aged 6–8 weeks, were obtained from the animal facility of the Federal University of Vale do São Francisco (UNIVASF). The animals were housed in a temperature-controlled environment (23 °C–25 °C) under a 12 h/12 h light-dark cycle with free access to standard chow and tap water prior experimentation. All experimental procedures were conducted in accordance with the guidelines of the Brazilian College of Animal Experimentation (COBEA) and were approved by the Ethics Committee on the Use of Animals (CEUA) of UNIVASF (protocol number 0004/230621). Animals were fasted for 18 or 24 h depending on the experimental protocol, with water provided *ad libitum* before testing.

### 2.6 Acute oral toxicity study

The acute oral toxicity study was conducted in accordance with the Organization for Economic Cooperation and Development (OECD) guideline 423 (OECD, 2002). Female Swiss mice (*n =* 3), individually housed, were used, in line with the guideline recommendation that females are generally more sensitive to toxic effects. Animals received a single oral dose of CEECs (2000 mg kg^-1^) by gavage. The control group received the vehicle only (3% Cremophor EL w/v, in saline) at the same administration volume (0.01 mL g^-1^).

After dosing, animals were observed at regular intervals during the first 24 h and subsequently once daily for 14 days to monitor behavioral changes, clinical signs of toxicity, and mortality. Food and water intake were recorded daily, and body weight was measured on days 0, 7, and 14.

At the end of the experimental period, animals were humanely euthanized by cervical dislocation. The heart, lungs, spleen, liver, stomach, and kidneys were excised, weighed, and examined macroscopically for pathological alterations.

### 2.7 Pharmacological assays

#### 2.7.1 Grouping and dosing of animals

Experimental groups, treatments, doses, and corresponding *in vivo* protocols are summarized in Table 1. Male mice were randomly allocated into groups of six. The negative control groups received the same vehicle used for CEECs administration. For the ethanol-induced gastric ulcer model, an additional normal control group (*n =* 3), which received vehicle instead of ethanol, was included to establish baseline gastric morphology. The vehicle used to solubilize CEECs as well the reference compounds used as positive controls in the experimental protocols consisted of 3% (w/v) Cremophor EL in saline. All treatments were administered orally, with the administered volume calculated according to OECD recommendations (0.01 mL g^-1^). The selection of the CEECs doses was based on the results of the acute oral toxicity study. Specific experimental procedures, treatment intervals, and outcome measures are detailed in the corresponding sections.

**TABLE 1:** Experimental groups, treatments, doses, and corresponding *in vivo* protocols used in the study. All treatments were administered orally, and the administered volume was calculated according to OECD recommendations (0.01 mL g^-1^).

Group	Treatment	Dose	Protocol
Negative control	Vehicle	0.01 mL g^-1^	All
Normal control	Vehicle	0.01 mL g^-1^	Ethanol-induced gastric ulcer
Positive control	Loperamide	10 mg kg^-1^	Gastric emptying
Positive control	Loperamide	20 mg kg^-1^	Castor oil- and magnesium sulfate-induced diarrhea
Positive control	Omeprazole	20 mg kg^-1^	Ethanol-induced gastric ulcer
Test group 1	CEECs	25 mg kg^-1^	All
Test group 2	CEECs	50 mg kg^-1^	All
Test group 3	CEECs	100 mg kg^-1^	All

#### 2.7.2 Evaluation of gastric emptying of liquid test meal

The effects of CEECs on gastric emptying were evaluated based on the protocol described by Silva et al. (2015), with experimental groups and dosing regimens summarized in Table 1. Thirty minutes after treatment, each animal received a liquid test meal consisting of 0.3 mL of a phenol red solution (0.5 mg mL^-1^) prepared in 5% glucose. Ten minutes after dye administration, animals were humanely euthanized by cervical dislocation, and the gastrointestinal tract was excised and separated into stomach and small intestine segments. Each segment was transferred to a vessel containing NaOH solution (0.1 N) for phenol red recovery. The contents were homogenized, and proteins were precipitated by the addition of 20% trichloroacetic acid. After centrifugation, an aliquot of the supernatant was alkalinized with NaOH solution (0.5 N). Phenol red content was determined by spectrophotometric analysis at 560 nm. Gastric emptying was calculated as the percentage of phenol red transferred from the stomach to the small intestine relative to the total amount of dye recovered from both segments.

#### 2.7.3 Antidiarrheal activity studies

##### 2.7.3.1 Castor oil-induced diarrhea model

CEECs antidiarrheal effects were evaluated using the castor oil-induced diarrhea model, adapted from the method described by Mehmood et al. (2011), with experimental groups and dosing regimens summarized in Table 1. One hour after treatment, diarrhea was induced by oral administration of castor oil (10 mL kg^-1^). Following castor oil administration, cages were inspected hourly to record the occurrence of diarrheal droppings. Results were expressed as median fecal output (g∙h^-1^) for each group.

##### 2.7.3.2 Magnesium sulfate-induced diarrhea model

CEECs antidiarrheal effects were also evaluated using the magnesium sulfate-induced diarrhea model, adapted from Uddin et al. (2005), with experimental groups and dosing regimens summarized in Table 1. Thirty minutes after treatment, diarrhea was induced by oral administration of magnesium sulfate (2 g kg^-1^). Following administration, cages were inspected hourly for 4 h to record the occurrence of diarrheal droppings. Results were expressed as mean fecal output (g) per animal over the 4 h observation period.

#### 2.7.4 Antiulcerogenic activity study

CEECs gastroprotective effects were evaluated using the ethanol-induced gastric mucosal injury model, adapted from Câmara Neto et al. (2022), with experimental groups and dosing regimens summarized in Table 1. One hour after treatment, acute gastric lesions were induced by intragastric administration of absolute ethanol (0.2 mL per animal) in all groups except the normal control group, which received vehicle instead of ethanol. One hour later, animals were humanely euthanized by cervical dislocation, and their stomachs were excised, opened along the greater curvature, and gently rinsed with saline. Each stomach was mounted between two Petri dishes, stretched, and photographed with a ruler for scale reference. Gastric lesions were measured using ImageJ software (Schneider et al., 2012), and the ulcerated area was expressed as a percentage of the total gastric surface.

### 2.8 Antibacterial activity studies

#### 2.8.1 Bacterial strains

Two bacterial strains commonly used as reference models in enteric disease research were selected, namely, *S. aureus* (ATCC 25923), obtained from the National Institute of Health Quality Control (INCQS/FIOCRUZ), and methicillin-resistant *Staphylococcus aureus* (MRSA, ATCC 33591) isolated from a clinical specimen. The choice of these strains was based on their relevance to enteric infections and toxin-mediated gastrointestinal disorders, in line with the traditional use of *C. sativa* roots for digestive ailments.

#### 2.8.2 Determination of the minimum inhibitory concentration (MIC)

MIC values were determined using the broth microdilution method, according to Alencar Filho et al. (2017) and Clinical and Laboratory Standards Institute (CLSI, 2025) guidelines. Sterile 96-well flat-bottom microplates were used. Serial one to one dilutions were prepared from a 25 mg mL^-1^ CEECs stock solution in 3% (w/v) DMSO and Müller-Hinton broth, producing final concentrations ranging from 12.5 to 0.19 mg mL^-1^. Each well received 10 µL of a 5 × 10^5^ CFU mL^-1^ bacterial inoculum. Plates were incubated for 18–24 h at 37 °C, after which 100 µL of a 1% w/v 2,3,5-triphenyltetrazolium chloride (TTC) solution was added to each well. The MIC was defined as the lowest extract concentration that completely inhibited bacterial growth. Sterile broth and gentamicin (12.5–0.19 mg mL^-1^) served as negative and positive controls, respectively. The final DMSO concentration did not exceed 3% (w/v).

#### 2.8.3 Determination of the minimum bactericidal concentration (MBC)

For MBC determination, 10 µL of each dilution was plated onto Müller-Hinton agar and incubated under identical conditions. The MBC was defined as the lowest concentration of extract showing complete absence of visible bacterial colonies.

### 2.9 Statistical analyses

Data normality was assessed using the Shapiro-Wilk test, and the selection of statistical tests was based on data distribution and assessment of variance homogeneity. Results of pharmacological and toxicological studies were expressed as the mean ± standard error of the mean (SEM) when data followed a normal distribution, whereas non-normally distributed data were expressed as median and interquartile range (IQR). Differences between means were evaluated using Student’s t-test or one-way analysis of variance (ANOVA) followed by Dunnett’s *post hoc* test. For non-normally distributed data, the Kruskal–Wallis test followed by Dunn’s test was applied. Statistical significance was set at *p* < 0.05. All analyses were performed in GraphPad Prism version 8.0.

## 3 Results

### 3.1 Total triterpenes quantification in the roots of *Cannabis sativa*

The total triterpene content of the CEECs, determined by spectrophotometric analysis, was 67.64 ± 5.39 µg LE∙mg^-1^.

### 3.2 Chromatographic analysis

**FIGURE 1:**
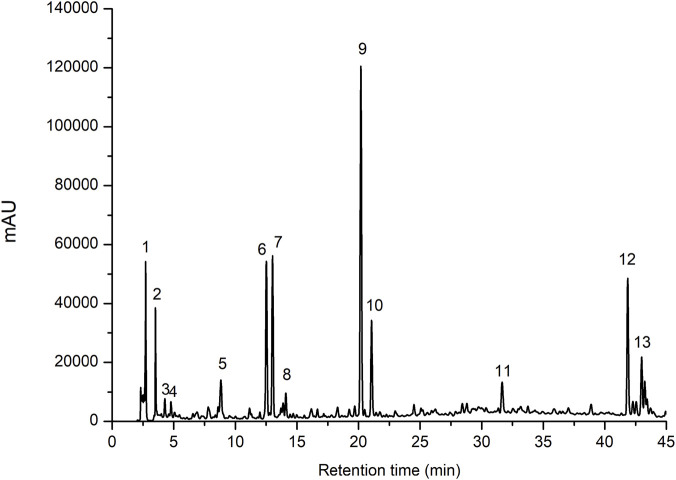
Representative high performance liquid chromatography (HPLC) chromatogram of the crude ethanolic extract of *Cannabis sativa* roots (CEECs). Separation was performed on a C18 reversed-phase column under gradient elution. Peaks were sequentially numbered according to retention time. Detection was carried out using a photodiode-array detector (PDA) at 280 nm.

The chromatographic conditions yielded a representative chromatogram of CEECs, comprising 13 distinct and well-resolved peaks, which were sequentially labeled (Figure 1). The corresponding retention times and maximum ultraviolet (UV) absorption wavelengths (λ_max_) of the detected peaks are summarized in Table 2.

**TABLE 2:** Retention times, ultraviolet absorption maxima, and relative peak areas of the peaks detected in the HPLC analysis of the crude ethanolic extract of *Cannabis sativa* roots (CEECs).

Peak number	Retention time (R_t_, min)	λ_max_ (nm)	Relative peak area (%)
1	2.72	-	4.02
2	3.51	-	2.15
3	4.26	264, 323	0.70
4	4.77	277, 362, 375	2.12
5	8.82	205, 266, 353	7.66
6	12.52	207, 225, 309	4.54
7	13.02	222, 292	4.64
8	14.10	292, 317	3.54
9	20.19	210, 222, 290	15.78
10	21.05	217, 292, 317	4.59
11	31.67	286, 318	36.95
12	41.85	201, 311	6.74
13	42.98	222, 288, 310	5.75

Ultraviolet absorption maxima were obtained by photodiode array detection. Relative peak areas were calculated by normalizing individual peak areas to the total integrated chromatographic area.

**FIGURE 2:**
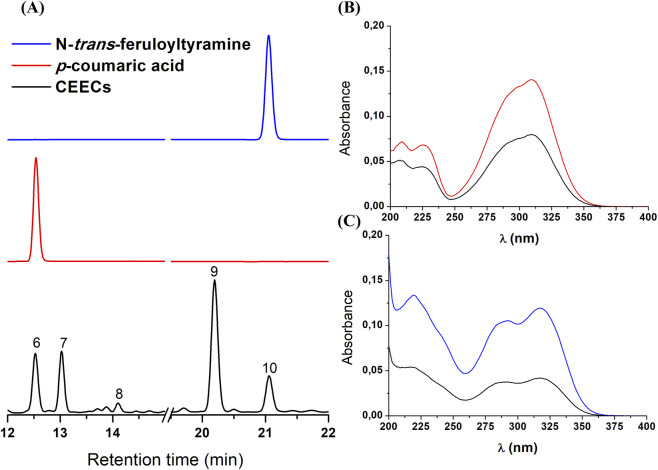
Chromatographic comparisons between the crude ethanolic extract of *Cannabis sativa* roots (CEECs) and reference standards analyzed under identical HPLC conditions. **(A)** Representative chromatograms of CEECs and the reference standards *p*-coumaric acid and N-*trans*-feruloyltyramine, with numbered peaks in the CEECs chromatogram corresponding to the peaks described in Table 1. **(B)** Ultraviolet absorption spectra obtained by photodiode array detection comparing peak six of CEECs with the *p-*coumaric acid standard. **(C)** Ultraviolet absorption spectra obtained by photodiode array detection comparing peak 10 with the N-*trans*-feruloyltyramine standard. Identification was based on the correspondence of retention times and UV absorption profiles.

Peak identification was performed by comparison of retention times and UV absorption profiles obtained by photodiode array detection with those of reference standards analyzed under identical conditions. Based on these criteria, peak 6 with a retention time of 12.52 min was identified as *p*-coumaric acid, and peak 10 with a retention time of 21.05 min was identified as N-*trans*-feruloyltyramine. Representative chromatograms and UV spectral comparisons with the corresponding standards are shown in Figure 2.

### 3.3 Toxicity study

Oral administration of CEECs at 2000 mg kg^-1^ did not induce clinical signs of toxicity, behavioral alterations, or mortality throughout the 14-day observation period. Macroscopic examination of the heart, lungs, spleen, liver, stomach, and kidneys revealed no visible pathological alterations.

**FIGURE 3:**
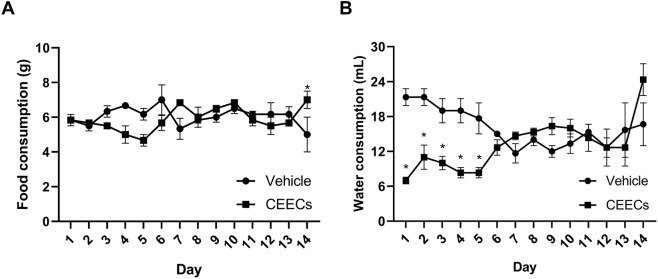
Effects of CEECs on food and water consumption during the acute oral toxicity study in mice. **(A)** Daily food consumption expressed as grams per animal. **(B)** Daily water consumption expressed as milliliters per animal. Data are presented as mean ± SEM (*n* = 3). *P < 0.05 versus control at the corresponding time points (Student’s t-test).

No statistically significant differences were observed between groups in overall food intake, total water intake, or body weight gain at the end of the experimental period (Table 3). However, analysis of daily water consumption revealed a transient reduction in the CEECs-treated group during the first 5 days following administration, when compared with the vehicle group (*P* < 0.05, Student’s t-test), as shown in Figure 3. This reduction was not accompanied by clinical signs of toxicity and was not sustained over the course of the experiment.

**TABLE 3:** Parameters evaluated during the acute oral toxicity study of CEECs in mice.

Parameter	Vehicle	CEECs
Food consumption (g/animal/day)	6.05 ± 0.14	5.89 ± 0.14
Water consumption (mL/animal/day)	16.0 ± 0.87	13.1 ± 1.17
Body weight at day 0 (g)	28.67 ± 1.09	32.17 ± 0.93
Body weight at day 7 (g)	29.00 ± 1.76	32.83 ± 1.20
Body weight at day 14 (g)	30.50 ± 1.32	33.17 ± 1.92
Body weight changes between days 0 and 14	1.83 ± 0.73	1.00 ± 1.04

### 3.4 Pharmacological assays

#### 3.4.1 Effect on gastric emptying

**FIGURE 4:**
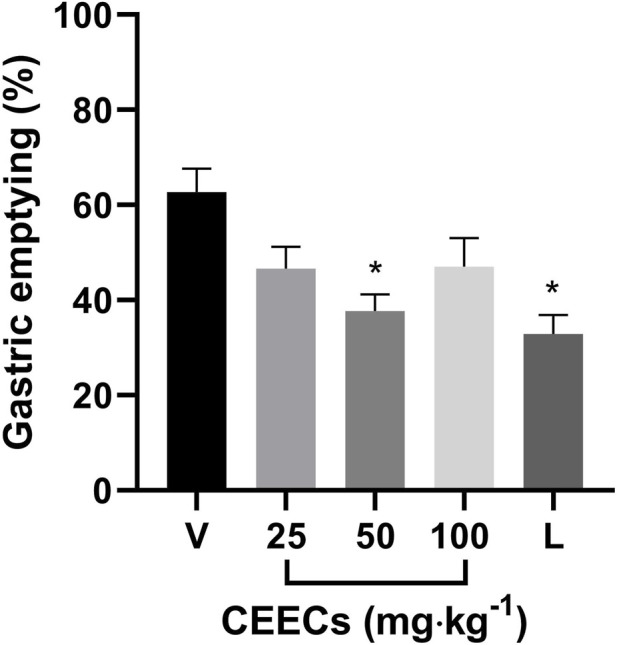
Effects of CEECs on gastric emptying of a liquid test meal in mice. Gastric emptying was evaluated 10 minutes after administration of a phenol red-labeled liquid test meal. V, vehicle; L, loperamide (10 mg kg^-1^). Data are presented as mean ± SEM. *P < 0.05 versus V (one-way ANOVA followed by Dunnet’s *post hoc* test).

Gastric emptying was evaluated 10 minutes after administration of a dye-labeled liquid test meal. As shown in Figure 4, mice treated with vehicle emptied 62.68% ± 4.94% of the gastric content. Treatment with CEECs resulted in emptying values of 46.64% ± 4.58%, 37.68% ± 3.56%, and 47.04% ± 6.00% at doses of 25, 50, and 100 mg kg^-1^, respectively. Loperamide (10 mg kg^-1^) reduced gastric emptying to 32.91% ± 3.95%.

One-way ANOVA revealed a significant effect of treatment on gastric emptying, F (4, 24) = 5.647, *P* = 0.0024 (Supplementary Table S2). Dunnett *post hoc* test showed that CEECs at 50 mg kg^-1^ (*P* = 0.0033) and loperamide (*P* < 0.001) significantly reduced gastric emptying compared with the vehicle group, whereas the doses of 25 and 100 mg kg^-1^ did not differ significantly (Supplementary Table S3).

#### 3.4.2 Effect of CEECs on diarrhea

The effects of CEECs on diarrhea were evaluated using castor oil-induced and magnesium sulfate-induced diarrhea models, with fecal output measured as the primary endpoint.

In the castor oil-induced diarrhea model, the fecal output was evaluated only in the first hour following castor oil administration, as this was the only time interval in which CEECs exhibited a measurable effect. No significant differences were observed in subsequent hours, and therefore only first-hour data are presented.

**FIGURE 5:**
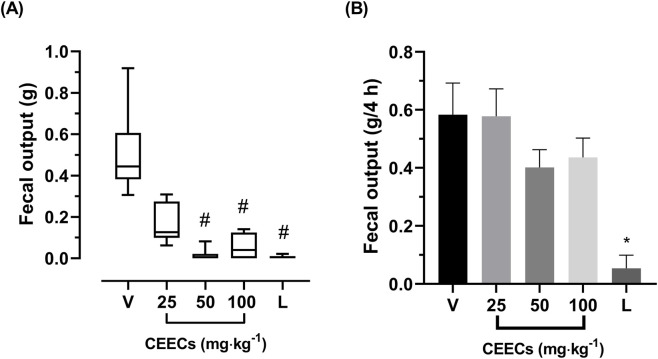
Effects of CEECs on the fecal output in experimental models of diarrhea in mice. **(A)** Castor oil-induced diarrhea model. Data are presented as median (IQR). **(B)** Magnesium sulfate-induced diarrhea model. Data are presented as mean ± SEM. V, vehicle; L, loperamide (20 mg kg^-1^). ^#^ *P* < 0.05 versus V (Kruskal–Wallis test followed by Dunn’s *post hoc* test). *P < 0.05 versus V (one-way ANOVA followed by Dunnett’s *post hoc* test).

The vehicle-treated group showed a median value of 0.44 g (0.22 g), as shown in Figure 5A. Treatment with CEECs at 25, 50, and 100 mg kg^-1^ resulted in median fecal outputs of 0.13 g (0.18 g), 0.00 g (0.02 g), and 0.04 g (0.12 g), respectively. The loperamide-treated group presented a median fecal output of 0.00 g (0.01 g). Statistical analysis using the Kruskal–Wallis test revealed a significant difference among groups (H = 22.98, df = 4, *P* < 0.001; Supplementary Table S4). Post hoc analysis with Dunn’s test showed that CEECs at 50 mg kg^-1^ (*P* < 0.001), CEECs at 100 mg kg^-1^ (*P* = 0.0233), and loperamide (*P* < 0.001) significantly reduced fecal output compared with the vehicle-treated group (Supplementary Table S5).

In the magnesium sulfate-induced diarrhea model, the vehicle-treated group presented a mean fecal output of 0.58 ± 0.11 g, as shown in Figure 5B. Treatment with CEECs at doses of 25, 50, and 100 mg kg^-1^ resulted in mean fecal outputs of 0.58 ± 0.09 g, 0.40 ± 0.06 g, and 0.44 ± 0.07 g, respectively. The loperamide-treated group presented a mean fecal output of 0.05 ± 0.05 g. One-way ANOVA revealed a statistically significant effect of treatment on fecal output, F (4, 22) = 8.419, *P* < 0.001 (Supplementary Table S6). However, *post hoc* analysis using Dunnett’s test indicated that only the loperamide-treated group differed significantly from the vehicle-treated group (*P* < 0.001; Supplementary Table S7).

#### 3.4.3 Effect of CEECs on gastric ulcers

**FIGURE 6:**
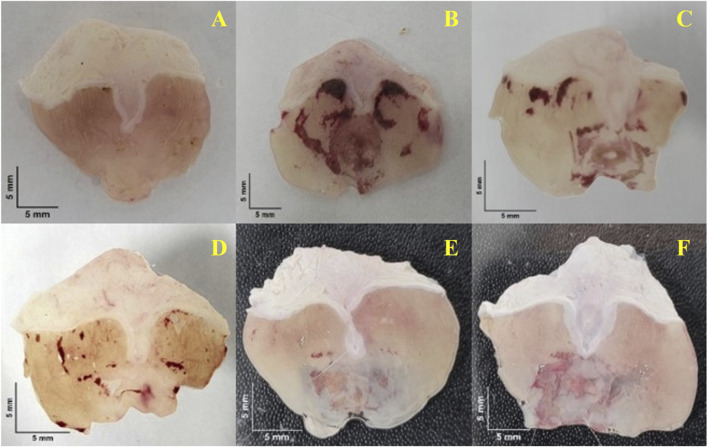
Representative macroscopic images of gastric mucosa from mice subjected to the ethanol-induced gastric ulcer model. **(A)** Normal group; **(B)** Ulcer control (vehicle-treated); **(C–E)** CEECs at 25, 50, and 100 mg kg^-1^; **(F)** Omeprazole at 20 mg kg^-1^. Scale bar = 5 mm.

As shown in Figure 6A, neither the gavage procedure nor the vehicle administration caused visible gastric injury in the stomachs of normal animals. In contrast, ethanol administration in the remaining groups produced evident mucosal damage characterized by hyperemia and hemorrhagic spots (Figures 6B–F).

**FIGURE 7:**
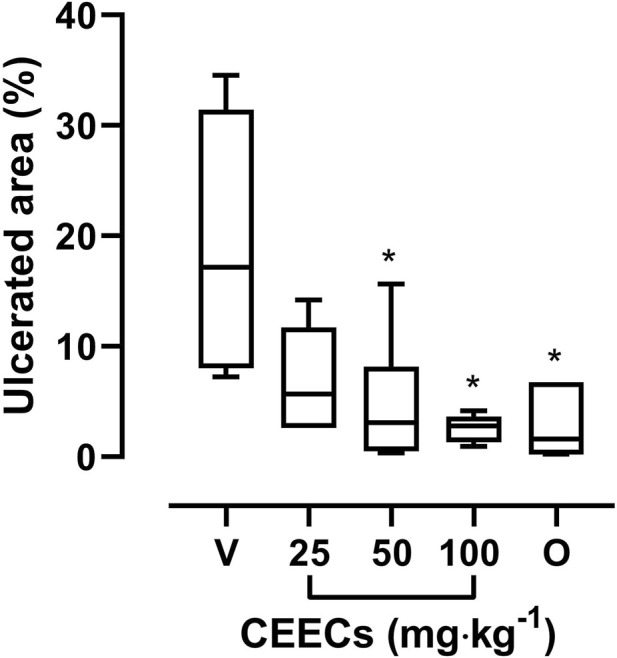
Effects of CEECs on ethanol-induced gastric injury in mice. Data are expressed as median (IQR). V, vehicle (ulcer control); O, omeprazole (20 mg kg^-1^). *P < 0.05 versus V (Kruskal–Wallis test followed by Dunn’s *post hoc* test).

Figure 7 shows the effect of CEECs on ethanol-induced gastric injury. The ulcer control group presented a median ulcerated area of 17.18% (23.41%). Treatment with CEECs reduced the extent of mucosal injury, with median ulcerated areas of 5.68% (9.11%), 2.89% (4.06%), and 2.81% (2.33%) at doses of 25, 50, and 100 mg kg^-1^, respectively. Treatment with Omeprazole (20 mg kg^-1^) resulted in a median ulcerated area of 1.62% (6.53%).

A Kruskal–Wallis test indicated a significant difference among groups (H = 13.75, df = 4, *P* = 0.0081; Supplementary Table S8). Dunn’s *post hoc* test showed that CEECs at 50 mg kg^-1^ (*P* = 0.0484), at 100 mg kg^-1^ (*P* = 0.0164), and omeprazole (*P* = 0.0045) significantly reduced the ulcerated area compared with the ulcer control group (Supplementary Table S9), whereas the dose of 25 mg kg^-1^ did not reach statistical significance.

### 3.5 Antibacterial studies

The antibacterial activity of CEECs was evaluated against 2 *S. aureus* strains. The MIC and MBC values are summarized in Table 4. CEECs exhibited identical MIC values for both strains. In contrast, the MBC values were higher than the MICs and exceeded the highest concentration tested, indicating the absence of bactericidal activity within the evaluated concentration range. Gentamicin, used as a positive control, showed MIC and MBC values below the lowest concentration tested for both strains.

**TABLE 4:** Minimum Inhibitory Concentration (MIC) and Minimum Bactericidal Concentration (MBC) of CEECs and gentamicin against *Staphylococcus aureus* strains.

Sample	*Staphylococcus aureus* (ATCC 25923)		MRSA (ATCC 33591)	
	MIC (mg·mL-1)	MBC (mg·mL-1)	MIC (mg·mL-1)	MBC (mg·mL-1)
CEECs	6.2	>12	6.2	>12
Gentamicin	<0.19	<0.19	<0.19	<0.19

## 4 Discussion

The present study investigated the chemical composition and pharmacological properties of *Cannabis sativa* L. roots, a plant part traditionally used to treat gastrointestinal disorders but still poorly explored from a pharmacological perspective. While most contemporary research on *C. sativa* has focused on the aerial parts and cannabinoid-rich tissues, experimental evidence validating the gastrointestinal use of the roots has been lacking.

Previous phytochemical studies have demonstrated that cannabis roots contain diverse classes of secondary metabolites, including monoterpenes, triterpenoids, alkaloids, amides, choline, and trace amounts of cannabinoids (Elhendawy et al., 2018; Lima et al., 2021; Huang et al., 2023). Among these constituents, pentacyclic triterpenes such as friedelin, epifriedelanol, β-amyrin, and related compounds have been consistently reported and are recognized for their broad spectrum of biological activities (Silva et al., 2014; Kornpointner et al., 2021; Huang et al., 2023). In this context, the present work provides an integrated experimental evaluation of the chemical profile, safety, and gastrointestinal pharmacological effects of an ethanolic extract obtained from *C. sativa* roots.

The spectrophotometric determination of total triterpene content confirmed the presence of this class of metabolites in CEECs at levels comparable to those previously reported for *C. sativa* roots (Jin et al., 2020). Although this approach does not allow structural discrimination among individual triterpenoids, it provides a useful global parameter for extract characterization and batch-to-batch comparison. To our knowledge, this is the first report describing the spectrophotometric quantification of total triterpenes in an ethanolic extract of *C. sativa* roots.

Chemical variability represents a major challenge in the development and standardization of herbal preparations, as it may arise from environmental conditions, processing methods, and storage parameters. In this context, chromatographic techniques play an important role in providing qualitative and semi-quantitative information on complex extracts (Elhendawy et al., 2018; Muyumba et al., 2021). In the present study, HPLC analysis yielded a chromatographic profile characterized by thirteen well-defined peaks, with *p*-coumaric acid and N-*trans*-feruloyltyramine identified based on retention times and UV absorption spectra.

The recurrent detection of these compounds in cannabis roots reported in independent studies (Menezes et al., 2021; Oh et al., 2022; Araújo et al., 2024) supports their consideration as potential chemical markers, although additional analytical validation would be required to establish a comprehensive chemical fingerprint suitable for regulatory purposes. One limitation of the present study is that the chemical characterization was performed on a single ethanolic extract obtained form plant material seized in a specific geographic region, which does not allow assessment of potential variability associated with cultivation conditions, genetic background, or post-harvest processing.

The acute oral toxicity evaluation indicated that CEECs presents a favorable safety profile under the tested conditions, supporting its use in subsequent *in vivo* pharmacological assays. These findings are consistent with previous reports describing the absence of detectable acute toxicity for aqueous extracts obtained from *C. sativa* roots (Lima et al., 2021). The transient reduction in water intake observed during the initial days following administration was not accompanied by clinical or behavioral alterations and was therefore not considered indicative of systemic toxicity.

Several gastrointestinal disorders have a prominent motility component. In this study, the effects of CEECs on gastrointestinal motility were evaluated using experimental models of gastric emptying and diarrhea. Gastric emptying is a coordinated physiological process involving neural, muscular, and interstitial elements, and its dysregulation is frequently associated with gastrointestinal disorders (Chikkamenahalli et al., 2020).

Under the experimental conditions employed, CEECs was associated with a reduction in gastric emptying at the dose of 50 mg kg^-1^, with an effect comparable in magnitude to that of loperamide, an antidiarrheal, spasmolytic, and constipating agent (Silva et al., 2015) used as a reference compound. Previous studies examining the effects of cannabis on gastrointestinal motility have focused predominantly on isolated cannabinoids or preparations derived from aerial plant parts (Pertwee, 2001; Camilleri and Zheng, 2023). The present findings extend these observations to a root-derived preparation, a plant part that has remained largely unexplored in experimental pharmacology.

Consistent with the observed effect on gastric emptying, the antidiarrheal activity of CEECs was evaluated using castor oil-induced and magnesium sulfate-induced diarrhea models. In the castor oil-induced model, ricinoleic acid released during castor oil hydrolysis disrupts water and electrolyte transport, leading to increased intestinal secretion and colonic contractions (Gilani et al., 2005). In the magnesium sulfate-induced model, magnesium sulfate inhibits water reabsorption and stimulates the release of cholecystokinin from the duodenal mucosa, resulting in increased intestinal motility and reduced fluid reabsorption (Uddin et al., 2005). In both assays, the fecal output was measured as the primary endpoint.

CEECs significantly reduced fecal output in the castor oil-induced model at doses of 50 and 100 mg kg^-1^, whereas no effect was observed in the magnesium sulfate model. Pre-treatment with loperamide significantly decreased diarrhea in both models. This context-dependent activity suggests that the antidiarrheal effect of CEECs is influenced by the underlying pathophysiological mechanisms involved in each model.

A previous study using an extract obtained from the aerial parts of *C. sativa* reported antidiarrheal effects in the same experimental model (Chda et al., 2023), with the authors proposing a predominant influence on intestinal secretory processes. Although that investigation involved a different plant part and extract composition, it provides a useful comparative framework for interpreting the selective activity observed for CEECs. Importantly, the present study does not allow mechanistic conclusions regarding secretory or motility pathways, and direct comparisons should be interpreted with caution.

Given the observed effects on motility and diarrhea models, the gastroprotective activity of CEECs was subsequently evaluated in an ethanol-induced gastric ulcer model. Ethanol-induced gastric injury is characterized by epithelial disruption, inflammatory infiltration, and impairment of mucosal defensive factors, including reductions in bicarbonate secretion, gastric mucus, and nitric oxide availability, as well as increased oxidative stress (Sistani Karampour et al., 2019; Chda et al., 2023). CEECs significantly reduced the extent of gastric injury at doses of 50 and 100 mg kg^-1^, while omeprazole, a classical proton pump inhibitor, exerted the expected protective effect. To our knowledge, these findings represent the first experimental evidence indicating antiulcerogenic activity for preparations derived from *C. sativa* roots.

Gastroprotective effects have previously been reported for cannabis extracts obtained from aerial parts. Abdel-Salam et al. (2015) demonstrated that an extract prepared from flowering tops containing 10% Δ^9^-THC dose-dependently reduced gastric mucosal damage induced by acidified aspirin and ethanol, in addition to attenuating gastric acid secretion and oxidative and inflammatory markers. More recently, Chda et al. (2023) evaluated extracts obtained from leaves and bracts after trichome removal in an attempt to reduce psychoactive components. Despite this modification, the extract also exhibited gastroprotective activity, which the authors associated with a complex phytochemical composition including cannabinoids, flavonoids, phytosterols, and terpenoids. Although these studies differ substantially from the present work in terms of plant part and extract composition, they provide a relevant reference indicating that gastroprotective effects have been described for cannabis formulations.

Beyond studies focused on cannabis preparations, a consistent association between pentacyclic triterpenoids and gastroprotective activity has been reported in diverse plant species. Antonisamy et al. (2015) demonstrated that friedelin, a pentacyclic triterpenoid also described in cannabis roots, exerted protective effects in experimental gastric injury models, accompanied by modulation of oxidative and inflammatory parameters. Similar gastroprotective activities have been described for triterpenoid-rich species such as *Maytenus robusta, Centella asiatica,* and *Glycyrrhiza* sp. (Cheng et al., 2004; De Andrade et al., 2008; Wang et al., 2017). In this context, Shen et al. (2025) reviewed experimental evidence indicating that oleanolic acid, another triterpenoid reported in cannabis roots, reduced gastric lesion areas in animal models of ulceration.

Taken together, these findings support the plausibility that triterpenoid-rich extracts may contribute to gastroprotective effects. However, mechanistic investigations were beyond the scope of the present study, and the specific molecular pathways underlying the observed activity remain to be elucidated.

The antibacterial evaluation revealed only weak inhibitory activity of CEECs against *S. aureus*, with no bactericidal effect within the tested concentration range. In contrast to cannabinoid-rich extracts from aerial parts, which have shown pronounced antibacterial activity (Chda et al., 2023; Mahou et al., 2024), the limited activity observed here is consistent with reports attributing modest antimicrobial effects of cannabis roots to non-cannabinoid constituents such as triterpenoids and sterols (Giselle et al., 2023; Gagné et al., 2024). Accordingly, antibacterial activity is unlikely to represent a major determinant of the gastrointestinal effects observed for CEECs and should be interpreted as complementary pharmacological information.

Although cannabinoids are reported to occur only in trace amounts in *C. sativa* roots, their potential contribution to the observed effects cannot be entirely excluded. However, the present study did not include quantitative cannabinoid analysis or evaluation of endocannabinoid signaling pathways. Therefore, any cannabinoid-mediated contribution warrants further investigation.

Overall, the present findings provide experimental support for the traditional use of *C. sativa* roots in the management of gastrointestinal disturbances, particularly diarrhea and gastric ulceration. By integrating chemical characterization, safety assessment, and *in vivo* pharmacological evaluation, this study contributes to the validation of an underexplored plant part within a contemporary pharmacological framework. Further studies are required to elucidate the molecular mechanisms involved and to refine the therapeutic potential of root-derived preparations.

## 5 Conclusion

From a chemical standpoint, this study established parameters that can support the quality control of materials derived from *Cannabis sativa* roots. These parameters include the total triterpene content and the chromatographic profile, which together provide a chemical basis for extract characterization and future standardization.

The crude ethanolic extract of cannabis roots (CEECs) exhibited modulatory effects on gastrointestinal motility and demonstrated gastroprotective activity under the experimental conditions employed. Specifically, CEECs delayed gastric emptying and reduced fecal output in the castor oil-induced diarrhea model. These effects were not associated with antibacterial activity, which was weak under the tested conditions.

Considering the known chemical composition of cannabis roots, the observed gastrointestinal effects may be associated with the presence of non-cannabinoid constituents, including pentacyclic triterpenes, which have been previously linked to antidiarrheal and gastroprotective activities in other plant species. In addition, CEECs attenuated ethanol-induced gastric ulceration, consistent with the recognized antiulcerogenic potential of triterpenoid-rich preparations.

Overall, this study provides experimental support for the traditional use of cannabis roots in the management of diarrhea and gastric discomfort. To our knowledge, such pharmacological effects have not been previously demonstrated for preparations derived specifically from *C. sativa* roots. Nevertheless, further studies are required to isolate the bioactive constituents and to elucidate the mechanisms underlying these effects, thereby supporting the rational development of standardized, non-psychoactive formulations for gastrointestinal disorders.

## Data Availability

The original contributions presented in the study are included in the article/Supplementary Material, further inquiries can be directed to the corresponding author.
