# Supplementary material for: Cannabis sativa L. roots extract modulates gastrointestinal motility and ameliorates ethanol-induced gastric ulcers in animal models
Source: Front Pharmacol. 2026 Jan 30;17:1743428. doi: 10.3389/fphar.2026.1743428 (PMC12900725; doi:10.3389/fphar.2026.1743428)
Supplement: Supplementary file 1 [file Supplementaryfile1.docx]

Supplementary Material

# Supplementary Tables

## Spectrophotometric quantification of total triterpenes

Table S1. Analysis of variance (ANOVA) for the linear regression model used in the spectrophotometric quantification of total triterpenes.

| **Source** | **df** | **SS** | **MS** | **F** |
| --- | --- | --- | --- | --- |
| Regression | 1 | 6.5331 | 6.5331 | 3779.2561 |
| Residual | 13 | 0.0224 | 0.0017 |  |
| Total | 15 | 6.5555 |  |  |

## Gastric Emptying Assay

**Table S2.**  One-way ANOVA for the gastric emptying assay.

| **Source** | **SS** | **df** | **MS** | **F** | ***P*** |
| --- | --- | --- | --- | --- | --- |
| Treatment | 2948 | 4 | 737.1 | 5.647 | 0.0024 |
| Residuals | 3133 | 24 | 130.5 |  |  |

**Table S3**. Post hoc analysis (Dunnett's test) results for the gastric emptying experiment.

| **Comparison** | **Mean**  **difference** | **95% CI**  **of difference** | **Standard Error** | **t** | **Adjusted *P*** |
| --- | --- | --- | --- | --- | --- |
| V vs. CEECs 25 mg∙kg^-1^ | 16.04 | -1.229 to 33.31 | 6.596 | 2.4314 | 0.0740 |
| V vs. CEECs 50 mg∙kg^-1^ | 25.00 | 7.731 to 42.27 | 6.596 | 3.7897 | 0.0033 |
| V vs. CEECs 100 mg∙kg^-1^ | 15.64 | -1.629 to 32.91 | 6.596 | 2.3707 | 0.0838 |
| V vs. Loperamide 20 mg∙kg^-1^ | 29.76 | 11.65 to 47.87 | 6.918 | 4.3020 | 0.0009 |

## Castor oil-induced diarrhea model

Table S4 - Kruskal-Wallis test for the castor oil-induced diarrhea model.

| **Factor** | **Statistic (H)** | **df** | ***P*** |
| --- | --- | --- | --- |
| Treatment | 22.98 | 4 | < 0.001 |

**Table S5**. Post hoc analysis (Dunn’s test) for the castor oil-induced diarrhea experiment.

| **Comparison** | **Mean rank difference** | **Adjusted *P*** |
| --- | --- | --- |
| V vs. CEECs 25 mg∙kg^-1^ | 6.833 | 0.6721 |
| V vs. CEECs 50 mg∙kg^-1^ | 18.50 | 0.0008 |
| V vs. CEECs 100 mg∙kg^-1^ | 13.67 | 0.0233 |
| V vs. Loperamide 20 mg∙kg^-1^ | 20.17 | 0.0002 |

## Magnesium sulfate-induced diarrhea model

**Table S6.** One-way ANOVA for the magnesium sulfate-induced diarrhea model.

| **Source** | **SS** | **df** | **MS** | **F** | ***P*** |
| --- | --- | --- | --- | --- | --- |
| Treatment | 1.054 | 4 | 0.2635 | 8.419 | 0.0003 |
| Residuals | 0.6886 | 22 | 0.03130 |  |  |

**Table S7**. Post hoc analysis (Dunnett's test) results for magnesium sulfate-induced diarrhea model.

| **Comparison** | **Mean**  **difference** | **95% CI**  **of difference** | **Standard Error** | **t** | **Adjusted *P*** |
| --- | --- | --- | --- | --- | --- |
| V vs. CEECs 25 mg∙kg^-1^ | 0.0057 | -0.2879 to 0.2992 | 0.1119 | 0.0506 | > 0.9999 |
| V vs. CEECs 50 mg∙kg^-1^ | 0.1816 | -0.1120 to 0.4751 | 0.1119 | 1.6227 | 0.3201 |
| V vs. CEECs100 mg∙kg^-1^ | 0.1472 | -0.1339 to 0.4283 | 0.1071 | 1.3742 | 0.4590 |
| V vs. Loperamide 20 mg∙kg^-1^ | 0.5298 | 0.2488 to 0.8109 | 0.1071 | 4.9459 | 0.0002 |

## Ethanol-induced gastric ulcer model

**Table S8.** Kruskal-Wallis test for the ethanol-induced gastric ulcer model.

| **Factor** | **Statistic (H)** | **df** | ***P*** |
| --- | --- | --- | --- |
| Treatment | 13.75 | 4 | 0.0081 |

**Table S9.** Post hoc analysis (Dunn’s test) for the ethanol-induced gastric ulcer model.

| **Comparison** | **Mean rank difference** | **Adjusted *P*** |
| --- | --- | --- |
| V vs. CEECs 25 mg∙kg^-1^ | 7.167 | 0.5250 |
| V vs. CEECs 50 mg∙kg^-1^ | 14.50 | 0.0484 |
| V vs. CEECs 100 mg∙kg^-1^ | 14.30 | 0.0164 |
| V vs. Omeprazole 20 mg∙kg^-1^ | 15.50 | 0.0045 |

# Supplementary Figures


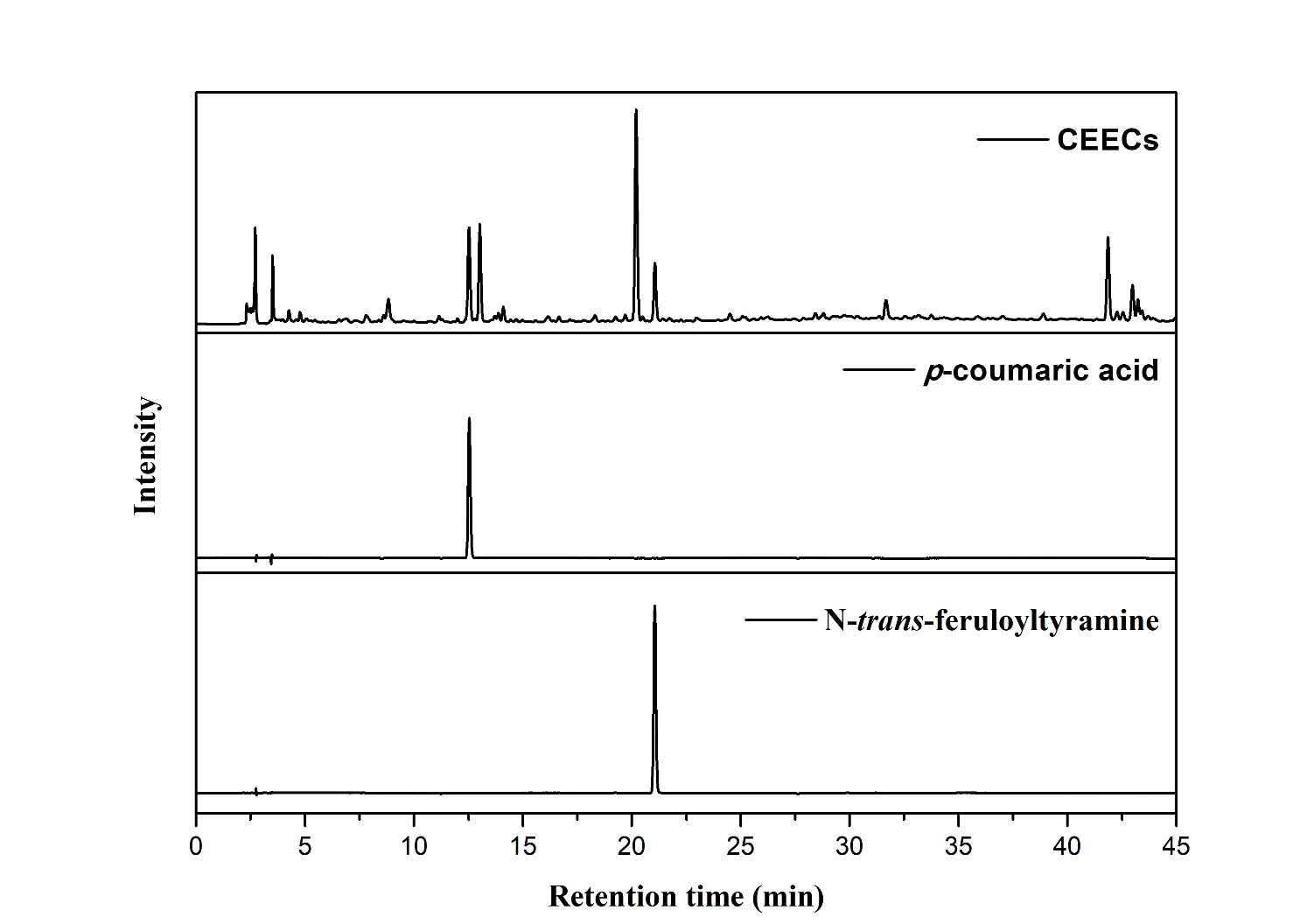


**Supplementary** **Figure** **1.** Original HPLC chromatograms of the crude ethanolic extract of *Cannabis* *sativa* roots (CEECs) and of the individual reference standards analyzed under identical chromatographic conditions. The chromatograms correspond to the full, uncropped data used to generate the representative sections shown in Figure 2 of the main manuscript.
